# Report of Case of Delivery, Complicated by Fibroid Tumor

**Published:** 1893-05

**Authors:** J. T. Crofford

**Affiliations:** Memphis, Tenn.


### REPORT OF CASE OF DELIVERY, COMPLICATED BY FIBROID TUMOR.

BY. J. T. CROFFORD, M. D., MEMPHIS, TENN.*

The woman, a primipara, was six months pregnant and had been in labor six days. The uterus could not empty itself on account of the fibroid growth encasing the whole cervical canal, preventing its dilatation. An arm of the tumor also projected in front of the mouth of the uterus, mechanically interfering with the passage of the contents of the organ. The pregnant uterus was high up in the abdomen and occupied the left side. The tumor was lower down and to the right of the median line. The diagnosis was of course obscure. The case was seen one hundred miles away in the country. The doctor was sent for for the purpose of doing a Cæsarian section, which would have required the supplement of a Porro, owing to the presence of sepsis. The poor equipment at hand caused him to remove the patient to his private sanitarium, which is located in Memphis. Arrangements were made for the operation, but the woman was so extremely exhausted that the doctor concluded to make a desperate attempt at delivery from

* Read before Tennessee State Medical Society, at Nashville, April 12th, 1893.

below, before subjecting so weak a woman to so formidable an operation. She was anæsthetized and placed in the lithotomy position. By making great pressure upon the vaginal portion of the tumor the doctor finally succeeded in somewhat pushing it aside, then by means of traction upon the womb it was brought somewhat lower. The cervical canal was forcibly dilated in spite of the surrounding band of the fibroid growth. The hand was next introduced and one foot of the foetus grasped and the delivery proceeded with. A trained assistant using the Crede method upon the outside, the placenta was finally delivered. Under hypodermics of strychnia and other stimulants the patient was rallied, became more comfortable and for a time progressed more favorably. The sepsis, however, continued in spite of daily uterine irrigations. Seventeen days later, although the patient was quite weak, the abdomen was opened with the idea of removing the offending growth, and if necessary the uterus as well. It was after the abdomen was laid open that the diagnosis was made and the relation of the uterus and tumor was first fully understood. The adhesions were so great however, that it was not thought that the patient could stand the breaking up of these with the addition of a hystero-myomotomy. The uterine appendages were removed, hoping to cause a shrinkage of the tumor. The condition of the patient did not seem to be made worse by the operation. The uterine irrigations were kept up. Six days later septic symptoms became still more formidable, the temperature going as high as 106 degrees, and the pulse to 145 beats per minute. Upon investigation the vaginal portion of the tumor as well as the interior of the uterine canal was found to be in a sloughing condition. The patient was again anæsthetized and placed in the same position as before, when the doctor by means of a knife and scissors removed large sections of the lower portion of the tumor, seared it with a thermo-cautery, then curetted and irrigated the uterine canal and packed the same with gauze. For three days she improved. This procedure was gone through with some half dozen times at intervals of three days until almost the whole of the tumor and a large portion of the uterus were removed. The patient finally made a complete recovery.

The doctor further stated that whilst this case teaches that it is not wise to apply the popular radical measures of the day to all such cases irrespective of the conditions existing, yet he emphasises the fact that the successful termination of this case should not be construed as a superiority of the measures adopted over the Porro operation. But upon the contrary he had no doubt that the Porro operation in this class of cases, in the hands of an experienced operator, offers safety beyond the measures here adopted, and would have gladly been utilized in this case had the patient been seen in time, or had her condition at any time thereafter justified it. The measures adopted as above given were the only ones that in his opinion, offered any reasonable hope of a successful termination in this particular case.

